# A Guide to a Pharmacist-Led Pharmacogenetic Testing and Counselling Service in an Interprofessional Healthcare Setting

**DOI:** 10.3390/pharmacy10040086

**Published:** 2022-07-19

**Authors:** Céline K. Stäuble, Chiara Jeiziner, Anna Bollinger, Florine M. Wiss, Martin Hatzinger, Kurt E. Hersberger, Thomas Ihde, Markus L. Lampert, Thorsten Mikoteit, Henriette E. Meyer zu Schwabedissen, Samuel S. Allemann

**Affiliations:** 1 Pharmaceutical Care, Department of Pharmaceutical Sciences, University of Basel, 4056 Basel, Switzerland; chiara.jeiziner@unibas.ch (C.J.); a.bollinger@unibas.ch (A.B.); florine.wiss@unibas.ch (F.M.W.); kurt.hersberger@unibas.ch (K.E.H.); markus.lampert@unibas.ch (M.L.L.); s.allemann@unibas.ch (S.S.A.); 2 Institute of Hospital Pharmacy, Solothurner Spitäler AG, 4600 Olten, Switzerland; 3 Biopharmacy, Department of Pharmaceutical Sciences, University of Basel, 4056 Basel, Switzerland; h.meyerzuschwabedissen@unibas.ch; 4 Psychiatric Services Solothurn, Solothurner Spitäler AG, Faculty of Medicine, University of Basel, 4503 Solothurn, Switzerland; martin.hatzinger@spital.so.ch (M.H.); thorsten.mikoteit@spital.so.ch (T.M.); 5 Institute of Psychiatry, Spitäler Frutigen Meiringen Interlaken AG (fmiAG), 3800 Unterseen, Switzerland; thomas.ihde@spitalfmi.ch

**Keywords:** pharmaceutical care, clinical pharmacy, medication review, pharmacy service, pharmacogenomics, personalized medicine, hospital pharmacy, community pharmacy, primary care, secondary care

## Abstract

Genetic predisposition is one factor influencing interindividual drug response. Pharmacogenetic information can be used to guide the selection and dosing of certain drugs. However, the implementation of pharmacogenetics (PGx) in clinical practice remains challenging. Defining a formal structure, as well as concrete procedures and clearly defined responsibilities, may facilitate and increase the use of PGx in clinical practice. Over 140 patient cases from an observational study in Switzerland formed the basis for the design and refinement of a pharmacist-led pharmacogenetics testing and counselling service (PGx service) in an interprofessional setting. Herein, we defined a six-step approach, including: (1) patient referral; (2) pre-test-counselling; (3) PGx testing; (4) medication review; (5) counselling; (6) follow-up. The six-step approach supports the importance of an interprofessional collaboration and the role of pharmacists in PGx testing and counselling across healthcare settings.

## 1. Introduction

In clinical practice, patients show individual responses to pharmacotherapy. While some experience an adequate effect, others do not respond at all, and some suffer from unwanted adverse reactions or even severe toxicities when taking the same drug at the same dose. Amongst many others, one reason for this may be the patients’ individual genetic predisposition. On the one hand, genetic variation can impact drug response by altering the expression and/or activity of genes encoding the enzymes and transporters that are involved in drug absorption, distribution, metabolism, or excretion (ADME), potentially affecting pharmacokinetics. On the other hand, genes encoding drug targets can also show variations, which may alter their structure, expression or activity, potentially affecting pharmacodynamics [1]. As an illustration, the enzyme cytochrome P450 2D6 (CYP2D6), which is involved in the metabolism of over 25% of marketed drugs, exhibits a wide range of metabolic capacities across a population. This is in part due to several known genetic variants translating into normal, increased, reduced, or even lacking enzyme activity. These enzymatic activities are grouped into four major phenotypes: (i) normal metabolizers (normal activity, NM); (ii) ultra-rapid or rapid metabolizers (increased activity, UM/RM); (iii) intermediate metabolizers (reduced activity, IM); (iv) poor metabolizers (no activity, PM). CYP2D6 genetic variants were found to affect the pharmacokinetics of several substrate drugs and thereby the risk of experiencing adverse drug reactions or therapy failure [2]. Compelling evidence in this context led to the incorporation of pharmacogenetic (PGx) information on drug labels and even to the publication of international PGx dosing guidelines for multiple CYP2D6 substrates, including analgesics, antidepressants, neuroleptics, antiarrhythmics and antiemetics [3,4,5].

Hitherto, PGx testing has become increasingly applicable in clinical practice as it becomes more and more affordable, and as advances in digital technology enable the integration of PGx information into clinical decision support tools [6]. However, PGx is not the only factor influencing drug response. In particular, other non-genetic factors may affect individual drug response as well, including physiological factors (e.g., age, sex, organ function); environmental factors (e.g., drug–drug interactions (DDI), food–drug interactions, smoking); and behavioral factors (e.g., medication adherence) [7,8]. Notably, a patient that is found to be a CYP2D6 normal metabolizer, based on PGx testing, may thus become an intermediate or even poor metabolizer through the co-administration of a CYP2D6-inhibitor. This deviation from an individual’s genotype-predicted phenotype by non-genetic factors is considered a phenoconversion [9]. However, PGx assessments in clinical practice are often focused on drug–gene interactions (DGI) only, without considering the other factors that are potentially needed for a patient-individual evaluation and integration of PGx information. In these cases, if the prediction of enzyme function for pharmacokinetic estimations is challenging, therapeutic drug monitoring (TDM) can be a relevant addition to PGx testing [10]. Clinical pharmacists are trained to consider a wide range of factors influencing drug response when performing a medication review to address drug-related problems [11]. Thereby, pharmacogenetic information may offer an additional piece to complete the medication review puzzle, enhancing more comprehensive and individualized analysis and therapy recommendations.

A glance at clinical practice shows that the integration of PGx in clinical routine is still modest or often lacking. Barriers to the application of PGx are diverse, including restricted reimbursement of PGx tests, partially limited evidence from prospective clinical trials, as well as a lack of education of healthcare professionals [12]. There are numerous notions addressing prospective PGx evidence e.g., [13,14,15], and the education of healthcare professionals, e.g., [16,17]. However, only a limited number of publications address how a PGx service aiming for patient-individual therapy recommendations can be designed, refined and applied in a real-world multi-professional healthcare setting e.g., [18]. A recent survey of Dutch pharmacists, physicians and patients, participating in a pilot study for an outpatient PGx service, found that the unclear allocation of responsibilities between healthcare professionals was a major barrier to the implementation of the PGx service [19]. Defining a formal structure, as well as concrete procedures and clearly defined responsibilities, may facilitate and increase the implementation of PGx testing in clinical practice. Herein, we describe the design and the refinement of a pharmacist-led PGx service in an interprofessional setting.

## 2. Materials and Methods

### 2.1. Service Design

The planning of the service started with the selection of a commercial provider of pharmacogenetic analyses applicable to an intervention in pharmacy practice. After a comparison of several commercial providers, we selected a system that was originally developed for pharmacogenetic testing in pharmacy practice (Stratipharm^®^, humatrix AG, Pfungstadt, Germany). The system offers sampling by buccal swabs, analyzing a panel of clinically relevant genetic variants (Table S1) that not only reports the geno- or haplotypes, but also provides a sophisticated phenotypic interpretation that is relevant to most of the drugs that are currently available on the European market. Within this system, accredited healthcare professionals can be granted access to the genetic information by a patient-owned personal code. Interpretation of the genetic data for the impact on selected drugs is continuously updated based on currently available evidence and recommendations extracted from Pubmed (www.pubmed.ncbi.nlm.nih.gov) (accessed on 1 April 2022); PharmGKB (www.pharmgkb.org) (accessed on 1 April 2022); and CPIC (www.cpicpgx.org) (accessed on 1 April 2022), respectively. Moreover, we adapted the proposed procedure of a service that was published by the U-PGx (Ubiquitous Pharmacogenomics) project strategy [14] to the Swiss healthcare system. The adaptation was carried out based on the information that was obtained from stakeholders from different fields who were involved in PGx, including clinical pharmacists, clinical pharmacologists, epidemiologists, the Swiss federal commission for genetic testing and professional associations. The final service description in the Results section follows the recommendations of the TIDieR (template for intervention description and replication) checklist for better reporting of interventions [20]. 

### 2.2. Service Refinement

**Figure 1:**
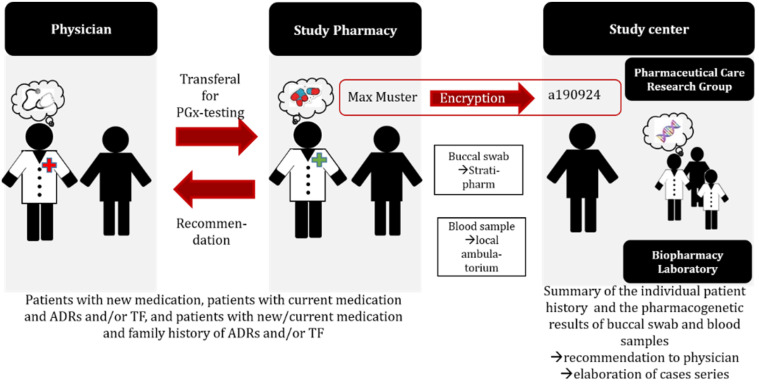
Study procedure of the observational case series study. ADR: adverse drug reaction; TF: treatment failure; PGx: pharmacogenetic.

The service, consisting of a comprehensive medication review [11] and supplemented with the individuals’ pharmacogenetic information to optimize drug selection and dosing, was originally started with single cases. After further standardization of the intervention, an observational case series study (ClinicalTrials.gov ID: NCT04154553) was launched. The primary objective of the case series was the compilation of case reports, where pharmacogenetic testing was applied to determine the hereditable component of the patient’s susceptibility to experience therapy failure (TF) and/or adverse drug reactions (ADR). Patients were recruited in the primary care setting, during hospitalization, or in ambulant hospital care. Eligible were adult patients either experiencing ADR or TF, or patients with a positive family history (of either); or patients with a planned/ new prescription for drugs that were known to be affected by genetic variants that influence their drug metabolism (pharmacokinetics) and/or the activity of the drug target (pharmacodynamics). Following the referral by the treating physician, the recruited patients underwent the process, as depicted in Figure 1. Individual cases from the series were published as case reports [21,22,23,24,25,26]. The work experience that was gathered within the observational case series study was used to further refine the PGx service over the duration of 3 years between 2019 and 2021, and was based on feedback from patients and treating physicians that was further elaborated in a mixed methods study [27].

## 3. Results

### 3.1. Service Description

**Figure 2:**
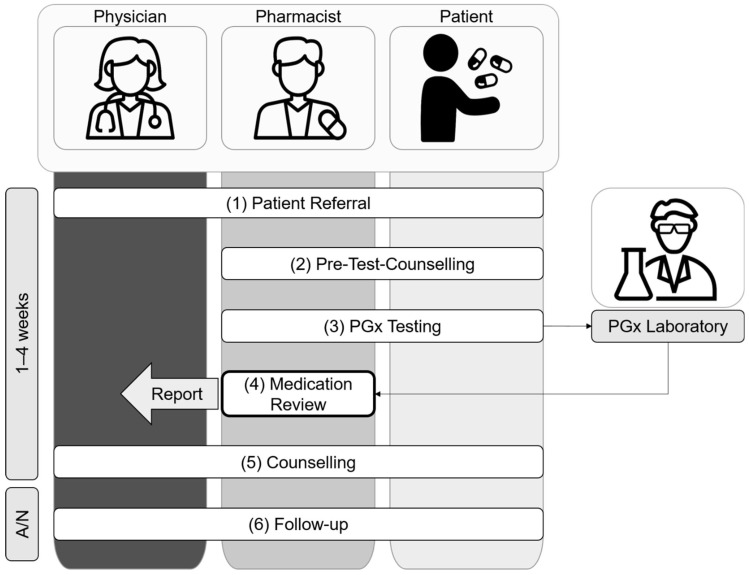
Overview of the pharmacist-led PGx service in an interprofessional setting.

The herein described service leads, within six steps, to the integration of pharmacogenetic information into a medication review by a pharmacist to serve as a rational basis for shared decision making, together with the treating physicians and the patient, in order to enable individualized pharmacotherapy optimization (Figure 2).

1. Patient Referral

Target patients have (a) experienced ADR and/or TF (reactive); (b) a planned new prescription or pharmacotherapy changes (preemptive); or (c) a family history of ADR and/or TF (preemptive). Patients are referred to the pharmacist-led PGx service (i) by their physician (general practitioner or specialist); (ii) based on own initiative (i.e., word-of-mouth); or (iii) by a pharmacist. In any case, treating physicians are informed and asked for their support for the planned pharmacist-led PGx service.

2. Pre-Test Counselling
  After referral to the pharmacist-led PGx service, the pharmacist and the patient meet face-to-face at the community/hospital pharmacy or at the hospital ward for a pre-test counselling visit to decide whether to proceed with PGx testing, following these steps:
  2.1 The pharmacist informs the patient about the goals, potential significance, and limits of PGx testing. In addition, the pharmacist answers any questions that the patient may have about PGx testing;
  2.2 The pharmacist performs a medication reconciliation and preliminary medication review of type 2a [11], using the Swiss polymedication check form [28] as an interview guide to (i) assess the patient’s current medication regimen; (ii) clarify the patient’s medication history, including experienced ADR and TF; (iii) identify any non-genetic drug related problems (e.g., drug–drug interaction, smoking, nutrition, renal and liver function, medication adherence, allergies). The pharmacist then clarifies any remaining ambiguities with family members or institutions providing care (e.g., home care, dispensing pharmacy, prescribing physician), provided that the patient agrees to do so. If urgent action is required due to identified drug-related problems (e.g., contraindications, need for therapeutic drug monitoring), the pharmacist immediately consults with the treating physician;
  2.3 The pharmacist decides whether to proceed with PGx testing based on the information that is available from the patient interview (2.2.). More precisely, there must either be pharmacogenetic recommendations available (e.g., CPIC guidelines) or a rationale from the drug’s metabolism for potential DGIs, for at least one substance or drug class that is indicated as suspicious. Substances are classified as conspicuous, e.g., either due to ADR and/or TF (reactive approach), or when considered for planned treatments (preemptive approach);
  2.4 The pharmacist collects the patient’s written informed consent for PGx testing. A copy of the signed informed consent is given to the patient. The pharmacist ensures that any questions the patient may have are answered. If the patient needs more time to decide, the further procedure may be postponed.
3. PGx Testing

The pharmacist collects a swab of the patient’s oral mucosa and ships it to the designated and approved PGx laboratory together with the signed informed consent (2.4). The PGx laboratory provides the pharmacist with the analyzed results from PGx testing (e.g., information about genetic variants and corresponding phenotype interpretation, processing time for Stratipharm^®^—max. 7–10 working days) and an online clinical decision support tool to check for DGIs.

4. Medication Review

The goal of the medication review process is (a) to detect drug-related problems and (b) to recommend specific medication changes or interventions, in order to optimize the patient’s pharmacotherapy to better meet his needs, and by this to ultimately improve health outcomes. Therefore, the pharmacist performs a structured evaluation of the patient’s past, current and planned medication, considering the available genetic and non-genetic information (2.2 and 2.3). To support this evaluation, the pharmacist consults (i) the PGx laboratory’s clinical decision support tool and the pharmGKB database (www.pharmgkb.org) (accessed on 1 April 2022) to assess DGIs; (ii) the summary of product characteristics and a drug interaction database (mediQ, www.mediq.ch) (accessed on 1 April 2022) to assess drug–drug interactions (DDI) and other drug related problems; (iii) a quantitative prediction tool to assess drug–drug–gene interactions (DDGI) (www.ddi-predictor.org) (accessed on 1 April 2022), combining the assessment results from (i) and (ii) (e.g., phenoconversion). Finally, the pharmacist prepares a written report with patient-specific recommendations and sends it to the treating physician.

5. Counselling

The pharmacist and/or treating physician communicate the PGx test results (3) and the medication review conclusions (4) to the patient in a face-to-face visit, phone call or video conference. The setting of the counselling is chosen based on the preferences of the patient and/or physician. In a process of shared decision making, the pharmacotherapy is adapted or additional laboratory analyses are initiated (e.g., therapeutic drug monitoring).

6. Follow-up

The pharmacist actively follows up with the patient one and six months after the counselling (5) to answer any further questions the patient may have and to assess the need for further counselling. The pharmacist offers the physician (i) follow-up counselling for further questions regarding the PGx test results, and (ii) an update of the medication review; for instance, in the case of major medication changes or shifts of variable non-genetic factors (e.g., renal function).

### 3.2. Service Refinement

The population of the case series observational study, which formed the basis for the PGx service refinement, consisted of 142 mainly female (66%) patients with a median age of 52 (IQR = 40–63) years. Around 60% of the patients were referred to the PGx service by a medical specialist doctor and about the same proportion was enrolled in the primary care setting (community pharmacy). A majority of the included patients had a main diagnosis of a mental or behavioral disorder (ICD-10 = F, 61%). The number of prescribed medicines reached a median of 6 (IQR = 4–9) per person, resulting in a majority of patients with polypharmacy (≥5 prescribed medicines, 62%), (Table 1).

The patients were included in the case series study to apply the PGx service based on a total of 549 suspected substances, which corresponded to a median of three suspected substances per patient (IQR 2–5). These were suspicious for DGIs due to clinically observed ineffectiveness (39%), ADR (40%) or both (5%). A smaller proportion gave cause to apply the PGx service preemptively due to planned new prescriptions (15%) or a family history of ADR and/or TF (0.6%). Slightly less than two-thirds of these suspected substances were eventually associated with any of the tested pharmacogenetic variations (n, 318; median, 2; IQR 1–3). The frequencies of genotype-predicted CYP2D6- and CYP2C19-phenotypes in our population correspond to the expected frequencies in the overall European population [29]. The patient-specific recommendations derived from the medication review by the pharmacists were implemented in about two-thirds of the cases that were followed up in both community (64%) and hospital pharmacy (66%) settings. The documented workload to perform the pre-test counselling, the medication review and the final counselling was on average 3 h per patient.

With the experiences from the case series, we refined the processes of patient referral (1); medication review (4); counselling (5); and follow-up (6) (Figure 2).

Initially, the patients were referred (Figure 2, step 1) to the intervention by their treating physicians who were informed about this opportunity in general practitioner quality circles or during hospital briefings. However, in pharmacy practice, drug-related problems are also directly addressed by pharmacists and/or patients during consultations and drug dispensing in the community pharmacy setting, or during interprofessional ward rounds and medication reconciliation in the hospital setting. Therefore, the pharmacists started to directly approach eligible patients. In a few cases, the patients approached the pharmacists through their own initiative due to word of mouth. In any case, treating physicians were informed and asked for their support for the planned pharmacist-led PGx service.

The case series increased the involved pharmacists’ and physicians’ knowledge and experience with pharmacogenetics, which influenced the medication review structure and content (Figure 2, step 4). Based on the experience, pharmacists started to supplement the medication review report with a concise overview of the patients’ pharmacogenetic profile and thereof predicted phenotypes, in order to facilitate the understanding about PGx information and to enable the application of these results to future drug-related problems and questions. Furthermore, the pharmacists provided interpretations for the impact of predicted phenotypes on pharmacokinetics for substances without explicit pharmacogenetic guidelines whenever reasonable.

The counselling visit (Figure 2, step 5) was originally intended to take place only between the pharmacist and the patient. Some physicians however preferred to take part in the counselling or even conduct the counselling themselves to facilitate shared decision making. Therefore, we started to organize the counselling visits individually based on the patients’ and physicians’ preferences, so that the pharmacist and/or the physician were able to conduct the counselling visit with the patient based on the medication review that was provided by the pharmacist.

The follow-up (Figure 2, step 6) was primarily intended to evaluate the implementation of the pharmacists’ recommendations within the case series study. However, the follow-up was additionally appreciated by the involved pharmacists, physicians, as well as patients, and was thus adapted accordingly. Patients and physicians received the opportunity to clarify open questions and place further queries. Pharmacists were able to collect continuous feedback on their recommendations and to remind the patients about the lifelong impact of their pharmacogenetic makeup.

## 4. Discussion

We propose a pharmacist-led PGx service for interprofessional settings in both primary and secondary care. This service was designed for and refined within the heterogeneous Swiss healthcare system, consisting of 26 different cantonal systems. Therefore, we believe that this service may also be applied in the healthcare systems of other countries. For the adaptation of this service, we have had good experience in consulting a wide range of experts, including clinical pharmacologists and epidemiologists, who are experienced in the field of PGx.

Projects with pharmacists who are involved in pharmacogenetic testing have been described for distinct healthcare settings, from primary care to individual clinics e.g., [18,30,31]. Our goal was to develop step-by-step guidance to encourage the practical implementation of a pharmacist-led pharmacogenetic service across healthcare settings and with the inclusion of other healthcare professionals and patients. Our experiences with the case series study showed that this approach was feasible in different settings and across a diverse sample of patients. However, we would like to highlight several remaining challenges when implementing such a service in clinical practice.

First, the documented workload of patient counselling and conducting the medication review was on average 3 h per patient. This cumulation does not include administrative work to arrange the appointments or sample shipping, nor the time that is invested for follow-up visits etc. This raises the question of resource management and reimbursement of the provided services. For instance, in Switzerland, PGx testing requires in most cases a prescription from a specialized pharmacologist to ensure basic healthcare coverage. Moreover, the initiation of PGx testing is by Swiss law currently limited to physicians. The Swiss law on genetic testing in humans is currently under revision, considering a notion to enable pharmacists to initiate PGx testing [32]. From our experience in the case series study, pharmacists became aware of drug-related problems that were potentially associated with PGx in their daily practice. Pharmacists, as important points of contact for patients when it comes to drug-related problems, are ideally placed to include pharmacogenetic information in their assessment of medication therapies. Enabling pharmacists to initiate PGx testing might enhance its implementation in clinical practice as an interprofessional service to improve patient outcomes.

Second, we consider equal and strong interprofessional collaboration to be a key factor for the implementation of the proposed service. The service involves at least four parties, namely, a pharmacist, a physician, a PGx laboratory and the patient. However, depending on the individual setting and notably for multimorbid elderly patients, there might also be more parties involved, for instance, additional physicians (general practitioners and medical specialists), therapists, nurses or other caregivers such as family members. Having existing and trusting relationships with all the involved parties proved essential for the success of implementing the service. While in secondary care, already established collaborations between healthcare professionals may facilitate the implementation of the PGx service, our experience shows that this service is also feasible in primary care settings. This is reflected in the fact that pharmacists’ recommendations were implemented with equal frequency in both primary and secondary care settings (ca. 65%). Still, implementing such a PGx service in primary care can be associated with an increased effort to establish essential interprofessional relationships. One prerequisite for a beneficial interprofessional collaboration in PGx is the continuous education of the involved healthcare professionals. Lacking knowledge of PGx amongst healthcare professionals has been described as a barrier to the implementation of PGx in clinical practice [12]. To address this, we have developed a blended-learning continuous educational program for pharmacists based on our work experience from the case series study. In the future, we plan to also include physicians, which may enhance interprofessional collaboration from the very start [16].

Third, the lack of digital data exchange between healthcare providers hinders communication and data sharing. So far, Switzerland lacks a consistent e-health strategy to overcome this barrier. Improved digital networks, considering data security, could enhance the continuous use of PGx information across healthcare settings and professions. Notably, germline genetic information has a lifelong validity. To overcome the large heterogeneity of data management systems, the U-PGx Consortium has adopted a so-called Safety-Code card system for their Europewide clinical trial (PREPARE). This personal card includes a basic overview of the individual’s PGx profile and a QR-code to access a web-based decision support tool with individual PGx dosing recommendations. The Safety-Code card allows for easy sharing of the genetic information between healthcare providers and empowers the patient to decide who can access their data [14]. Apart from the accessibility of PGx information, digital interfaces are also important to facilitate access for healthcare providers to other relevant non-genetic information. As mentioned before, PGx information should be analyzed in context with non-genetic information, including co-medication and medication history. Therefore, a nationwide electronic health record (EHR) system would be of great benefit to ensure access for all healthcare providers to both genetic, as well as non-genetic health data. One of the early adopters of such an EHR system is Estonia, where a central digital repository provides access to an individual’s lifelong medical history, including PGx information (https://e-estonia.com/) (accessed on 1 April 2022).

## 5. Conclusions and Outlook

Our proposed PGx service was feasible in an interprofessional and heterogeneous healthcare setting. Over 60% of our recommendations were implemented and we recorded a continuous referral of over 140 patients for 3 years. Our experience shows that a PGx service within an interprofessional setting needs a clear structure and assignment of tasks. Access to (electronic) patient data and remuneration for the service remain important barriers to the implementation in clinical practice. Moreover, follow-up studies are warranted to assess the impact (e.g., clinical outcome, cost-effectiveness) of such a PGx medication review intervention in selective patient cohorts. Based on our experiences, we selected psychiatric patients with major depression for a first ongoing outcome study [13]. Finally, pharmacists as specialists in pharmacotherapy and important points of contact for drug-related problems are ideally placed to initiate PGx testing and support other healthcare professionals with patient-specific medication reviews.

## Figures and Tables

**Table 1:** Demographics of the observational case series study.

Characteristic	Category	Number (%) or Median (IQR)
Subjects, n	-	142
Age (years), median (IQR)	-	52 (40–63) (min. 18, max. 88)
Gender, n (%)	Female	93 (65.5)
	Male	49 (34.5)
Referring party, n (%)	Medical specialist	92 (64.8)
	General practitioner	25 (17.6)
	Pharmacist	25 (17.6)
Enrollment setting, n (%)	Community pharmacy	85 (59.9)
	Hospital pharmacy	57 (40.1)
Main diagnosis, n (%)	Mental and behavioral disorders (ICD-10: F)	86 (60.6)
	Diseases of the musculoskeletal system and connective tissue (ICD-10: M)	30 (21.1)
	Diseases of the circulatory system (ICD-10: I)	15 (10.6)
	Other *	11 (7.8)
Number of prescribed medicines, median (IQR)	-	6 (4–9)
Polypharmacy (≥5 prescribed medicines), n (%)	-	92 (62.2)

* ICD-10: C (neoplasms); -E (endocrine, nutritional and metabolic diseases); -G (diseases of the nervous system); -R (symptoms, signs and abnormal clinical and laboratory findings, not elsewhere classified); -U (codes for special purposes); or -Z (factors influencing health status and contact with health services).

## Data Availability

The data (incl. genetic data) presented in this study are available on request from the corresponding author. The data are not publicly available for ethical and privacy reasons.

## Supplementary Materials

The following supporting information can be downloaded at: https://www.mdpi.com/article/10.3390/pharmacy10040086/s1, Table S1: Stratipharm^®^ (humatrix AG, Pfungstadt, Germany) SNPs and annotations.
