# Supplementary material for: A Guide to a Pharmacist-Led Pharmacogenetic Testing and Counselling Service in an Interprofessional Healthcare Setting
Source: Pharmacy (Basel). 2022 Jul 19;10(4):86. doi: 10.3390/pharmacy10040086 (PMC9326676; doi:10.3390/pharmacy10040086)
Supplement: Supplementary file 1 [file pharmacy-10-00086-s001.zip › pharmacy-1815538-supplementary.pdf]

Supplementary Material

Table S1. Stratipharm® (humatrix AG, Pfungstadt, Germany) SNPs and annotations

Gene	Chromosome	Annotation	Position	Amino acid replacement	Base
**ABCB1**	Chromosom 7q21.12	**rs1045642**	NM_000927.4:c.3435T>C	I1145I	T>C
**ABCB1**	Chromosom 7q21.12	**rs1128503**	NM_000927.4:c.1236T>C	G412G	T>C
**ABCB1**	Chromosom 7q21.12	**rs2032582**	NM_000927.4:c.2677G>A	A893T	G>A
**ABCB1**	Chromosom 7q21.12	**rs2032582**	NM_000927.4:c.2677G>T	A893S	G>T
**ABCB1**	Chromosom 7q21.12	**rs2032583**	NM_000927.4:c.2685+49T>C	-	T>C
**ABCG2**	Chromosom 4q22-q23	**rs2231142**	NM_004827.2:c.421C>A	Q141K	C>A
**ABCG2**	Chromosom 4q22-q23	**rs13120400**	NM_004827.2:c.1194+928A>G	-	A>G
**ABCG2**	Chromosom 4q22-q23	**rs17731538**	NC_000004.11:g.89055379G>A	-	G>A
**ADRB1**	Chromosom 10q24-q26	**rs1801252**	NM_000684.2:c.145A>G	S49G	A>G
**ADRB1**	Chromosom 10q24-q26	**rs1801253**	NM_000684.2:c.1165G>C	G389R	G>C
**ADRB2**	Chromosom 5q31-q32	**rs1042713**	NT_029289.11:g.9369367G>A	G16R	G>A
**ADRB2**	Chromosom 5q31-q32	**rs1042714**	NC_000005.9:g.148206473G>C	E27Q	G>C
**COMT**	Chromosom 22q11.21	**rs4680**	NM_000754.3:c.472G>A	V158M	G>A
**COMT**	Chromosom 22q11.21	**rs165599**	NM_000754.3:c.*522G>A	-	G>A
**COMT**	Chromosom 22q11.21	**rs4646316**	NM_000754.3:c.615+310C>T	-	C>T
**COMT**	Chromosom 22q11.21	**rs9332377**	NM_000754.3:c.616-367C>T	-	C>T
**COQ2**	Chromosom 4q21.23	**rs4693075**	NC_000004.11:g.84192168G>C	-	G>C
**COQ2**	Chromosom 4q21.23	**rs6535454**	NM_015697.7:c.894T>C	D298D	T>C
**CYP1A2**	Chromosom 15q24.1	**rs2069514**	NC_000015.9:g.75038220G>A	-	G>A
**CYP1A2**	Chromosom 15q24.1	**rs762551**	NC_000015.9:g.75041917C>A	-	C>A
**CYP2B6**	Chromosom 19q13.2	**rs8192709**	NM_000767.4:c.64C>T	R22C	C>T
**CYP2B6**	Chromosom 19q13.2	**rs28399499**	NM_000767.4:c.983T>C	I328T	T>C
**CYP2B6**	Chromosom 19q13.2	**rs3745274**	NM_000767.4:c.516G>T	Q172H	G>T
**CYP2C8**	Chromosom 10q24.1	**rs10509681**	NM_000770.3:c.1196A>G	K399R	A>G
**CYP2C8**	Chromosom 10q24.1	**rs11572080**	NM_000770.3:c.416G>A	R139K	G>A
**CYP2C8**	Chromosom 10q24.1	**rs1934951**	NG_007972.1:g.35707G>A	-	G>A
**CYP2C9**	Chromosom 10q24.1	**rs1799853**	NM_000771.3:c.430C>T	R144C	C>T
**CYP2C9**	Chromosom 10q24.1	**rs1057910**	NM_000771.3:c.1075A>C	I359L	A>C
**CYP2C9**	Chromosom 10q24.1	**rs9332131**	NM_000771.3:c.817delA	K273X	delA
**CYP2C9**	Chromosom 10q24.1	**rs7900194**	NM_000771.3:c.449G>A	R150H	G>A
**CYP2C9**	Chromosom 10q24.1	**rs28371685**	NM_000771.3:c.1003C>T	R335W	C>T
**CYP2C19**	Chromosom 10q24	**rs4244285**	NM_000769.1:c.681G>A	-	G>A
**CYP2C19**	Chromosom 10q24	**rs4986893**	NM_000769.1:c.636G>A	W212X	G>A
**CYP2C19**	Chromosom 10q24	**rs12248560**	NG_008384.1:g.4195C>T	-	C>T
**CYP2C19**	Chromosom 10q24	**rs28399504**	NM_000769.1:c.1A>G	M1V	A>G

**CYP2D6**	Chromosom 22q13.1	**-**	copy number variation	-	CNV
**CYP2D6**	Chromosom 22q13.1	**rs35742686**	NM_000106.4:c.775delA	-	delA
**CYP2D6**	Chromosom 22q13.1	**rs3892097**	NM_000106.4:c.506-1G>A	-	G>A
**CYP2D6**	Chromosom 22q13.1	**rs5030655**	NM_000106.4:c.454delT	-	delT
**CYP2D6**	Chromosom 22q13.1	**rs5030867**	NM_000106.4:c.971A>C	H324P	A>C
**CYP2D6**	Chromosom 22q13.1	**rs5030865**	NM_000106.4:c.505G>T	G169X	G>T
**CYP2D6**	Chromosom 22q13.1	**rs5030865**	NM_000106.4:c.505G>A	G169R	G>A
**CYP2D6**	Chromosom 22q13.1	**rs5030656**	NM_000106.5:c.841_843delAAG	K281del	delAAG
**CYP2D6**	Chromosom 22q13.1	**rs1065852**	NM_000106.4:c.100C>T	P34S	C>T
**CYP2D6**	Chromosom 22q13.1	**rs201377835**	NM_000106.5:c.181-1G>C	-	G>C
**CYP2D6**	Chromosom 22q13.1	**rs28371706**	NM_000106.4:c.320C>T	T107I	C>T
**CYP2D6**	Chromosom 22q13.1	**rs59421388**	NM_000106.4:c.1012G>A	V338M	G>A
**CYP2D6**	Chromosom 22q13.1	**rs28371725**	NM_000106.4:c.985+39G>A	-	G>A
**CYP3A4**	Chromosom 7q21.1	**rs2740574**	NG_000004.3:g.135607G>A	-	G>A
**CYP3A4**	Chromosom 7q21.1	**rs2242480**	NM_017460.5:c.1026+12G>A	-	G>A
**CYP3A5**	Chromosom 7q21.1	**rs776746**	NM_000777.3:c.219-237G>A	-	G>A
**DPYD**	Chromosom 1p22	**rs3918290**	NM_000110.3:c.1905+1G>A	-	G>A
**DPYD**	Chromosom 1p22	**rs72549303**	NM_000110.3:c.1898delC	-	delC
**DPYD**	Chromosom 1p22	**rs72549309**	NM_000110.3:c.298delTinsTCAT	-	delTinsTCAT
**DPYD**	Chromosom 1p22	**rs55886062**	NM_000110.3:c.1679T>G	I560S	T>G
**DPYD**	Chromosom 1p22	**rs67376798**	NM_000110.3:c.2846A>T	D949V	A>T
**DPYD**	Chromosom 1p22	**rs2297595**	NM_000110.3:c.496A>G	M166V	A>G
**GNB3**	Chromosom 12p13	**rs5443**	NM_002075.2:c.825C>T	S275S	C>T
**GSTP1**	Chromosom 11q13.2	**rs1695**	NM_000852.3:c.313A>G	I105V	A>G
**HLA-A**	Chromosom 6p21.3	**rs1061235**	NM_002116.7:c.*66A>T	-	A>T
**HLA-A**	Chromosom 6p21.3	**rs1633021**	NC_000006.12:g.29779092T>C	-	T>C
**HLA-B**	Chromosom 6p21.3	**rs3909184**	NM_005803.2:c.724-507C>G	-	C>G
**HLA-B**	Chromosom 6p21.3	**rs2395029**	NM_006674.3:c.*568T>G	-	T>G
**HLA-B**	Chromosom 6p21.3	**rs2844682**	NC_000006.11:g.30946148G>A	-	G>A
**HMGCR**	Chromosom 5q13.3-q14	**rs17238540**	NM_000859.2:c.2457+117T>G	-	T>G
**HMGCR**	Chromosom 5q13.3-q14	**rs17244841**	NM_000859.2:c.451-174A>T	-	A>T
**HTR2A**	Chromosom 13q14-q21	**rs6311**	NC_000013.10:g.47471478C>T	-	C>T
**HTR2A**	Chromosom 13q14-q21	**rs6313**	NM_000621.3:c.102C>T	S34S	C>T
**HTR2A**	Chromosom 13q14-q21	**rs7997012**	NM_000621.3:c.614-2211T>C	-	T>C
**HTR2A**	Chromosom 13q14-q21	**rs9316233**	NC_000013.10:g.47433355C>G	-	C>G
**HTR2A**	Chromosom 13q14-q21	**rs6314**	NC_000013.10:g.47409034G>A	H368Y	G>A
**IFNL3**	Chromosom 19q13.13	**rs8099917**	NC_000019.9:g.39743165T>G	-	T>G
**IFNL3**	Chromosom 19q13.13	**rs12979860**	NC_000019.9:g.39738787C>T	-	C>T
**ITPA**	Chromosom 20p	**rs1127354**	NM_181493.1:c.43C>A	P32T	C>A

**NAT2**	Chromosom 8p22	**rs1801280**	NM_000015.2:c.341T>C	I114T	T>C
**NAT2**	Chromosom 8p22	**rs1799930**	NM_000015.2:c.590G>A	R197Q	G>A
**NAT2**	Chromosom 8p22	**rs1799931**	NM_000015.2:c.857G>A	G286E	G>A
**OPRM1**	Chromosom 6q24-q25	**rs1799971**	NM_000914.3:c.118A>G	N40D	A>G
**SLC19A1**	Chromosom 21q22.3	**rs1051266**	NM_194255.1:c.80A>G	H27R	A>G
**SLCO1B1**	Chromosom 12p12	**rs4149056**	NM_006446.4:c.521T>C	V174A	T>C
**SLCO1B1**	Chromosom 12p12	**rs11045819**	NM_006446.4:c.463C>A	P155T	C>A
**SLCO1B1**	Chromosom 12p12	**rs2306283**	NM_006446.4:c.388A>G	N130D	A>G
**SLCO1B1**	Chromosom 12p12	**rs4149015**	NG_011745.1:g.4195G>A	-	G>A
**TPMT**	Chromosom 6p22.3	**rs1800462**	NM_000367.2:c.238G>C	A80P	G>C
**TPMT**	Chromosom 6p22.3	**rs1800460**	NM_000367.2:c.460G>A	A154T	G>A
**TPMT**	Chromosom 6p22.3	**rs1142345**	NM_000367.2:c.719A>G	Y240C	A>G
**TPMT**	Chromosom 6p22.3	**rs1800584**	NM_000367.2:c.626-1G>A	-	G>A
**TPMT**	Chromosom 6p22.3	**rs12201199**	NM_000367.2:c.419+94T>A	-	T>A
**VKORC1**	Chromosom 16p11.2	**rs9923231**	NC_000016.9:g.31107689C>T	-	C>T
**VKORC1**	Chromosom 16p11.2	**rs7294**	NM_024006.4:c.*134G>A	-	G>A
**VKORC1**	Chromosom 16p11.2	**rs17708472**	NM_024006.4:c.173+525C>T	-	C>T
**VKORC1**	Chromosom 16p11.2	**rs2359612**	NM_024006.4:c.283+837T>C	-	T>C
**VKORC1**	Chromosom 16p11.2	**rs8050894**	NM_024006.4:c.283+124G>C	-	G>C
**VKORC1**	Chromosom 16p11.2	**rs9934438**	NM_024006.4:c.174-136C>T	-	C>T
